# Autism, spectrum or clusters? An EEG coherence study

**DOI:** 10.1186/s12883-019-1254-1

**Published:** 2019-02-14

**Authors:** Frank H. Duffy, Heidelise Als

**Affiliations:** 1 Department of Neurology, Boston Children’s Hospital and Harvard Medical School, Boston, USA; 2 Department of Psychiatry, Boston Children’s Hospital and Harvard Medical School, 300 Longwood Avenue, Enders 107, Boston, MA 02115 USA

**Keywords:** Autism spectrum disorder (ASD), Asperger’s syndrome (ASP), EEG coherence factors, Connectivity, NbClust, K-means, Hierarchical, Cluster analysis, Discriminant analysis

## Abstract

**Background:**

Autism prevalence continues to grow, yet a universally agreed upon etiology is lacking despite manifold evidence of abnormalities especially in terms of genetics and epigenetics. The authors postulate that the broad definition of an *omnibus* ‘spectrum disorder’ may inhibit delineation of meaningful clinical correlations. This paper presents evidence that an objectively defined, EEG based brain measure may be helpful in illuminating the autism spectrum versus subgroups (clusters) question.

**Methods:**

Forty objectively defined EEG coherence factors created in prior studies demonstrated reliable separation of neuro-typical controls from subjects with autism, and reliable separation of subjects with Asperger’s syndrome from all other subjects within the autism spectrum and from neurotypical controls. In the current study, these forty previously defined EEG coherence factors were used prospectively within a large (*N* = 430) population of subjects with autism in order to determine quantitatively the potential existence of separate clusters within this population.

**Results:**

By use of a recently published software package, NbClust, the current investigation determined that the 40 EEG coherence factors reliably identified two distinct clusters within the larger population of subjects with autism. These two clusters demonstrated highly significant differences. Of interest, many more subjects with Asperger’s syndrome fell into one rather than the other cluster.

**Conclusions:**

EEG coherence factors provide evidence of two highly significant separate clusters within the subject population with autism. The establishment of a unitary “Autism Spectrum Disorder” does a disservice to patients and clinicians, hinders much needed scientific exploration, and likely leads to less than optimal educational and/or interventional efforts.

## Background

The DSM-5 [1] summarizes that individuals on the autism spectrum exhibit problems involving interaction and communication with other individuals, show repetitive behaviors and restricted interests, and manifest behavior issues interfering with school, work, and/or multiple other life endeavors. The move from DSM-3 [2] to DSM-4 [3] and most recently DSM-5 [1] as diagnostic standard reflects a gradual condensation of a number of autism-related clinical entities under the rubric of Autism Spectrum Disorder (ASD). These include infantile autism, atypical autism, pervasive developmental disorder not otherwise specified or PDD-nos, and most recently Asperger’s syndrome [4]. This diagnostic “simplification” was welcomed by some yet quite concerning to others, as previously reviewed [5]. As suggested by Kienle et al. [6], “…the issue of an empirically reproducible and clinically feasible differentiation into subgroups must still be raised.” Indeed, in 2016, Pruett and Povinelli [7] published a wistful ‘Commentary’ in which they hypothesized that the usual rapid and automatic recognition of individuals on the autism spectrum resulted from our human “evolved sensitivity for species-typical ranges of social relating”. The authors further postulated that “social spacing”, “quality of eye contact”, and “timing of communicative exchange” constitute three primary variables and that they may form a recognizable set of “clusters” within the realm of human behavior.

A review of the literature from 1994 to 2018 reveals nine publications using cluster analysis to demonstrate quantitatively defined groupings within subjects diagnosed with autism spectrum disorder (ASD) [6, 8–15]. Eight papers used structured neurobehavioral assessments of various types [2, 3, 16–24] and one relied upon MRI data. Four studies reported solutions involving two clusters, one reported both two and three cluster solutions, one study reported a three cluster solution, two reported a four cluster solution, and one reported a five cluster solution.

It is notable, that all studies summarized above succeeded in identifying clusters. However, the number of underlying clusters identified varied, although the two cluster solution was noted most often. As an ensemble, the studies serve to suggest that ASD may well comprise a varying number of discrete sub-populations rather than exist on a continuum.

The varying number of clusters reported in these different studies may reflect the unique characteristics of the population under study, differing choices of variables selected to represent subjects and/or differing cluster methodologies utilized. The two most commonly used cluster methods, hierarchical and K-means algorithms rely upon apparent ‘satisfactory’ cluster separation by means of the clinical/neuropsychological difference between or among subjects within differing clusters. K-means clustering, hierarchical clustering, and combinations of these two fundamental methods, fail to determine quantitatively the optimal cluster number. For instance, the K-means approach to clustering “…requires users to specify the number of clusters to be generated. One fundamental question is: How to choose the right number of clusters” [25], p 39. Similarly, “…one of the problems with hierarchical clustering is that is does not tell us how many clusters there are…” [25], p 74.

The current study employed one of the first comprehensive software approaches to objectively establish cluster number, namely NbClust [25]. It is specifically designed to provide an objective means, i.e. independent of investigator choice, to identify the ‘optimal’ cluster number within a population.

Thus, the main goal of the current study was to determine the feasibility of delineating objective, EEG-based, clusters among children diagnosed with ASD. The underlying hope is that successful clustering might ultimately lead to better clinical, cost effective, specific diagnoses of subtypes of Autism and the creation of more specific interventions as well as a method to test the effectiveness of such interventions, be they pharmacological, behavioral or other. The multiplicity of potential approaches to clustering and their complexities have been succinctly reviewed by Jain et al. [26] and more recently by Charrad et al. [25]. Key issues that arise when employing cluster analysis are as follows: (1) What clustering technique to use (typical choices are K-means and hierarchical); (2) How many clusters to form (typically required prior to analysis initiation); and (3) How to determine the relative “significance” of resulting cluster configurations (internally by statistics and/or externally by association with one or more symptom complexes). The software package NbClust [25] addresses these three issues and was used in this study to elucidate the cluster structure within a large group of ASD subjects each represented by 40 previously derived [27] EEG-based coherence factors.

The main hypothesis of the study thus was that there are definable subgroups (clusters) of children within autism. It was hypothesized that children in one cluster will be different from children in other groups (clusters), and more similar among each other within a cluster, than across clusters. A secondary hypothesis states that EEG coherence is a productive means for the establishment of such stable clusters of children within the autism population.

## Methods

### Overview

1. Utilize for clustering 430 subjects with ASD, who had been studied previously for a different purpose [27].
2. Utilize as variables for clustering all 40 EEG coherence factors objectively generated previously in the differentiation of subjects with ASD and neurotypical control (CON) group subjects [27].
3. Determine the ASD cluster number within the 430 previously studied subjects with ASD by use of the recently developed NbClust software package [25, 28].
4. Compare NbClust results with independently used hierarchical and K-means clustering techniques.
5. For both hierarchical and K-means clustering algorithms use initial default parameters, and then initiate by use of NbClust all thirty available methods in order to determine the optimal cluster number, assessing up to 15 possible cluster outcomes by an objective ‘voting process’, which is part of NbClust [25].
6. Utilize the ‘voted’ as best outcome cluster configuration to identify each ASD subject’s cluster association/identity.
7. Evaluate the internal validity of the resulting clusters within multivariate factor space by using univariate and multivariate statistics (discriminant function analysis - DFA with jackknifing and split-half replication).
8. Explore by use of multigroup DFA the relationship among derived clusters and a neurotypical control (CON) population (not used for clustering). And.
9. In order to explore external cluster meaning, evaluate the relationship, i.e., multivariate positioning, of previously studied ASP subjects [5], who were not part of the ASD population used to form EEG-based factors and were not included in the clustering process.

### Subjects previously studied

The EEG coherence factor data used for this study were derived from a population of 984 previously studied 2 to 12 year old subjects [27]. Of these subjects, 430 were representatives of the Autism Spectrum Disorder group (ASD) and 554 constituted the neurotypical control group (CON).

As previously detailed [27], the *ASD population* had EEGs to rule out epilepsy, seen in up to 30% of certain ASD patients [12, 29]. ASD referrals for the prior study came from pediatric psychologists, psychiatrists, or neurologists at Boston Children’s Hospital (BCH) or from another Harvard associated teaching hospital. Diagnosis of ASD relied upon the DSM [1, 30] and/or ADOS [31, 32] criteria confirmed by clinical histories and evaluation. ASD exclusion criteria included: (1) Coexistent neurologic syndromes with autistic-like features, (2) Seizure disorders or epileptic encephalopathy (infrequent and/or isolated spikes did not cause exclusion); (3) Primary diagnoses of global developmental delay or dysphasia; (4) Clinical uncertainty as to the diagnosis of ASD; (5) Medication being taken at the time of study; (6) Any processes that might alter EEG change such as hydrocephalus, hemiparesis, or other syndromes often associated with abnormal brain development.

As also previously outlined [27], the *CON population* was selected from an extensive study pool archived by the BCH Developmental Neurophysiology Laboratory (DNL). CON subjects had been utilized as controls for numerous research projects over many years. CON subjects constituted a comparison group of children selected to be normally functioning yet avoiding creation of a ‘super-normal’ population. All CON group subjects were living at home with, considered normal by parents and identified as functioning within the normal range on standardized assessments from respective research studies. Previously delineated *CON exclusion criteria* included: (1) Diagnosed with or suspected of psychiatric or neurologic illness; (2) Abnormal neurological examination; (3) Seizure disorder (Rare EEG spikes were permitted); (4) Noted at study time to manifest autistic features; (5) Receiving medications.

### Summary of the EEG data collection protocol, the analytic methods previously utilized, and the prior study results

**Fig. 1:**
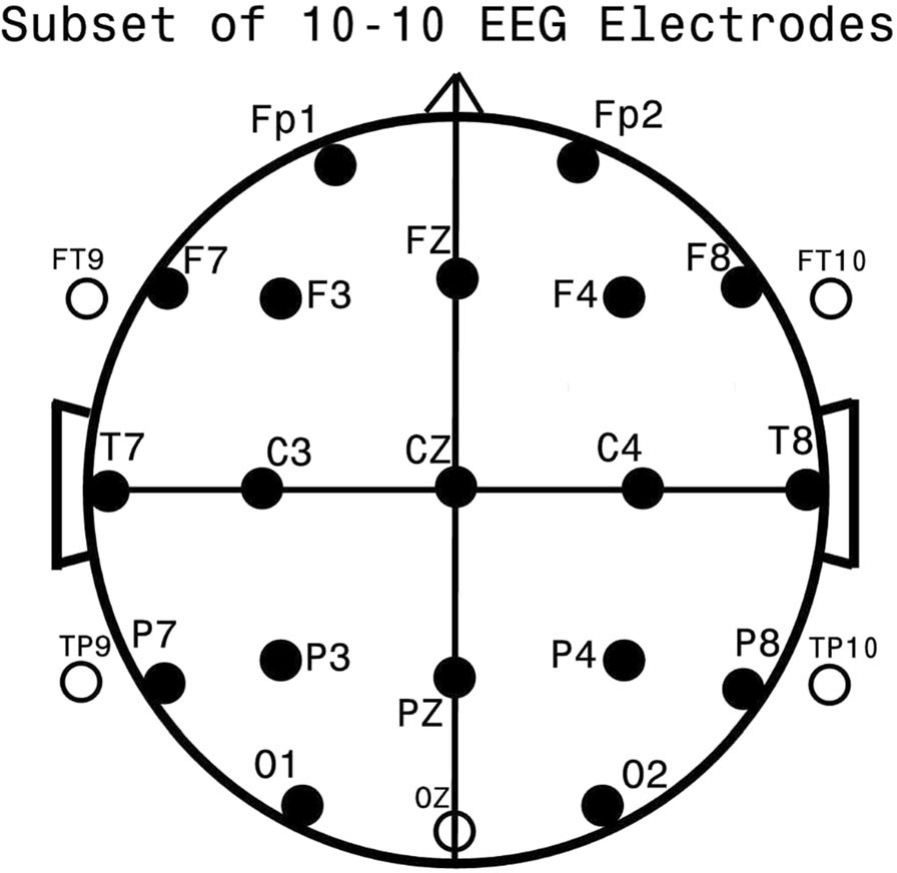
Standard EEG Electrode Names and Positions. Legend: Head in vertex view, nose above, left ear to left. EEG electrodes: Z: Midline: FZ: Midline Frontal; CZ: Midline Central; PZ: Midline Parietal; OZ: Midline Occipital. Even numbers, right hemisphere locations; odd numbers, left hemisphere locations: Fp: Frontopolar; F: Frontal; C: Central; T: Temporal; P: Parietal; O: Occipital. The standard 19, 10–20 electrodes are shown as black circles. An additional subset of five, 10–10 electrodes are shown as open circles. This figure was first published in a 2012 autism manuscript by the current authors [27] and is shown with permission of these authors and publisher, BMC Medicine

Data for all subjects were digitally recorded at BCH, in the resting awake state following placement of 24 gold cup electrodes (Fig. 1) with EEG filtered from 1 to 100 Hz at 256 Hz sampling rate. More recent data were recorded at a higher spatial density (128 channels) and temporal sampling rate (512 Hz). These data were software down-sampled to conform to the earlier recorded data as previously detailed [27]. From 8 to 20 min of artifact-free waking data were collected. As EEGs had been primarily collected to rule out epilepsy, these records usually contained additional time for the appearance of drowsiness and/or sleep as epileptiform discharges are often more frequent during these periods [33]. No subjects were included if EEG records were deemed diagnostic of or consistent with an underlying seizure disorder. Coherence analyses were restricted to waking epochs. Segments of EEG containing obvious artifacts were eliminated by visual inspection. Remaining eye blink and eye movement artifacts, often prominent even during the eye closed state, were removed by means of a source component technique [34, 35] implemented by the BESA™ software package. EEG data were analyzed in Laplacian montage [36–38] with coherence calculated [36] between all pairs of 24 electrodes (Fig. 1) in 16, two Hz spectral bands from 1 to 30 Hz resulting in 4416 unique spectral values per subject. Impact of any remaining eye blink and muscle artifact upon these coherence measures were removed by multiple regression using frontal slow delta and high frequency frontal-temporal EEG as indicators (used as independent variables in multiple regression) of residual eye and muscle artifact respectively [27, 39, 40].

Reduction of the 4416 coherence variables was managed by Principal Components Analysis (PCA). The first 40 factors accounted for 50.87% of the total variance. Age effects were removed from the 40 coherence variables generated on the total sample by regression of age at study. Factors remained statistically uncorrelated after age regression. These 40 factors were used to separate the CON from ASD groups by discriminant function analysis (DFA); results were highly significant (*p* < 0.0001) [27]. More importantly, when DFA was used in 10 randomly formed split half replications, the average ASD group classification success was 86.0% and for the CON group, 88.5%. For each split half replication, classification success was also highly significant (*p* < 0.0001). It was concluded that “…consistent differences exist between the CON and ASD groups” [27].

### Cluster analysis, current study

Clustering is a technique of “unsupervised learning” that partitions subjects/objects into groupings or “clusters” such that the subjects/objects within a cluster are more similar to others within the cluster than to subjects in other clusters. The NbClust cluster analysis program [25], within the extensive “R” analytic and display software packages [28, 41], was selected for the purpose of objective, unbiased estimation of the optimal number of clusters within a data set, a primary issue when performing cluster analysis. NbClust, a recent addition to the R programming and analyses software packages, provides 30 indices to determine the “best” number of clusters in a data set by objective, data-driven, “majority vote”. NbClust also provides [28] both hierarchical and K-means clustering as options. The 40 EEG coherence factors developed and described in a prior study [27] and the data from the prior ASD group were utilized as variables for cluster analysis in the current study.

Internal validation of clusters, once delineated, was assessed by three criteria: *First,* that a majority of the 40 factor values differed between clusters by T-test; *Second*, that the clusters differed among/between themselves by two-group discriminant function analysis (DFA - see below); and *Third*, when the CON group was added to the newly defined ASD clusters that the CON group and the created ASD clusters respectively remained separate by multi-group DFA. External cluster validation, in the absence of available, consistent neuropsychological and/or other domain-derived variables for the ASD subjects, was limited in the current study to the passive localization of previously evaluated [5] EEG coherence factor data from 26 ASP subjects within the multivariate space of the CON and ASD subject clusters.

### Discriminant function analysis (DFA) and other statistical procedures

All statistical analyses, aside from PCA and cluster analyses, utilized the BMDP2007™ software package [42]. Program 7 M (P7M) was used for the two and three group stepwise discriminant function analyses (DFA). P7M creates new canonical variables for maximal subject group separation. For a two group analysis one discriminant function is produced and for a three group analysis two discriminant functions are produced. DFA defines the significance of a group separation, summarizes the classification of each participant, and provides an approach for the prospective classification of individuals not involved in creation of the discriminant rule [43, 44]. In order to estimate prospective classification success, the jackknifing technique, also referred to as the leaving-one-out process, was utilized. By this method, a discriminant function is formed on all individuals but one. The left-out individual is subsequently classified. This initial left out individual is then folded back into the group (hence ‘jackknifing’), and a different individual is left out, a process which is repeated until all individuals have been left out and classified by a classification rule created on the non-left out subjects. The measure of classification success is then based upon a tally of the correct classifications of the left-out individuals. An alternative technique to estimate prospective classification success was also utilized, namely split-half replication. By means of a random number generator, internal to P7M, the entire population was randomly divided into a training-set and a test-set. Classification rules were generated on the training-set and evaluated in terms of classification on the corresponding test-set. Such split-half replication was repeated five times.

## Results

### Cluster creation

**Fig. 2:**
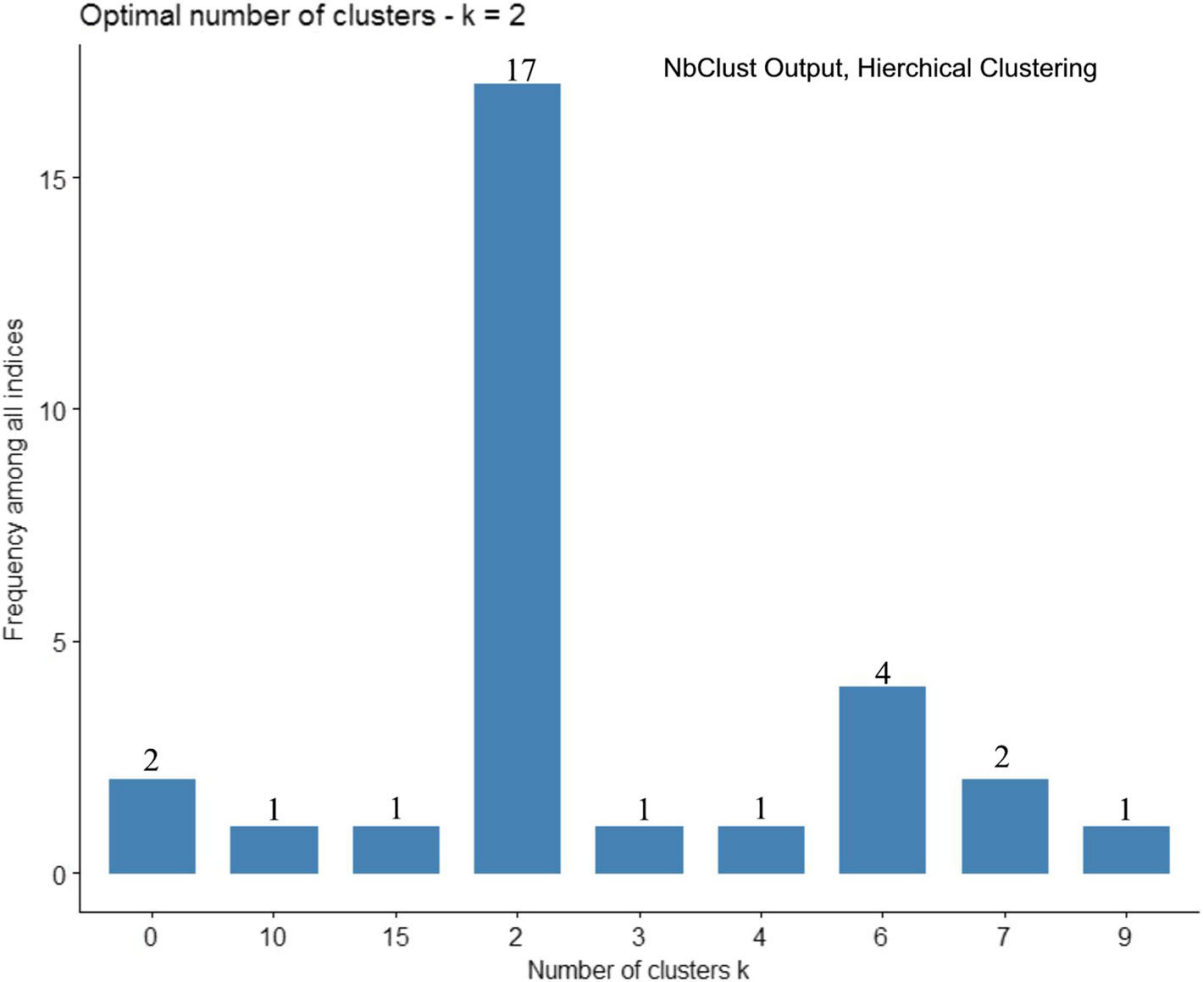
Optimal Cluster Number by Hierarchical Clustering and Program NbClust. Legend: NbClust produced histogram of up to 15 possible cluster groupings formed by Hierarchical clustering. Atop each vertical bar is the total number of the 30 indices used to estimate the optimal cluster grouping. Note that 17 of the 30 indices indicate the two cluster configuration as “optimal”. Cluster configurations never selected are omitted from the X axis as their frequency would be zero

**Fig. 3:**
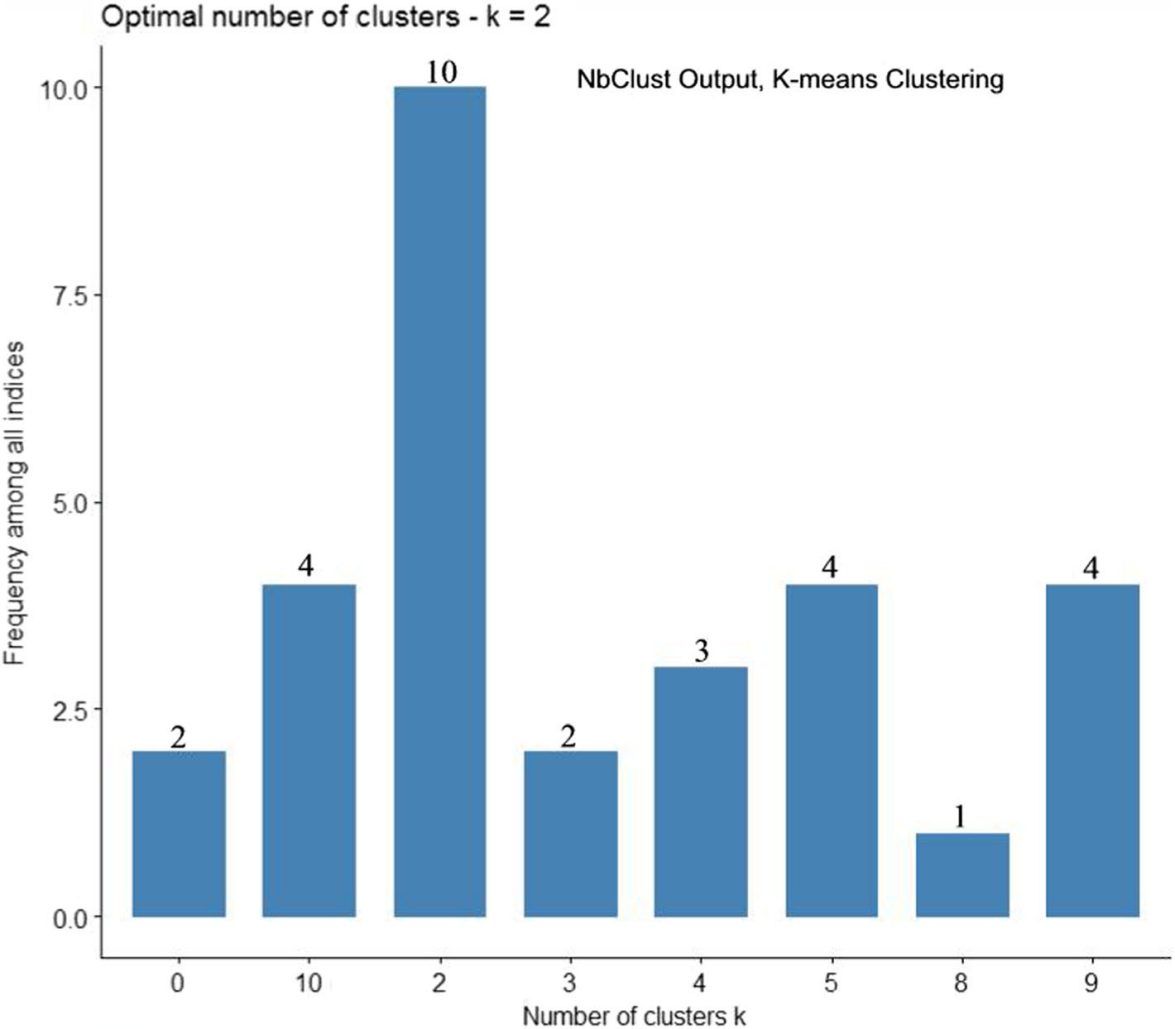
Optimal Cluster Number by K-means Clustering and Program NbClust. Legend: An NbClust produced histogram of up to 15 possible cluster groupings formed by K-means clustering. Atop each vertical bar is the total number of the 30 indices used to estimate the optimal cluster grouping. Note that 10 of the 30 indices indicate the two cluster configuration as “optimal”. Cluster configurations never selected are omitted from the X axis as their frequency would be zero

NbClust was performed on 430 ASD subjects represented by 40 factor variables [27] using the hierarchical clustering method and asking for the development of up to 15 clusters. Results are shown in histogram form, see Fig. 2. Note that 17 of the 30 assessments identified a 2-cluster solution and by the majority rule of the hierarchical approach this was determined the optimal cluster configuration. NbClust was repeated now using K-means clustering with results shown in Fig. 3. NbClust once more indicated as optimal the 2-cluster-configuration; 10 of the 30 assessments ‘voted’ for this outcome. Since both clustering methods chose the 2 cluster solution as optimal, this configuration was taken as most representative of the full ASD population. Moreover, as hierarchical clustering produced the more definitive 17 of 30 ‘vote’, the ‘best’ two cluster solution as formed from hierarchical clustering, was accepted. The first cluster, referred to as Cluster 1 or C1, comprised 169 subjects and the second cluster, referred to as Cluster 2 or C2, contained 261 subjects.

### Separation between clusters; factors and demographic variables

Table 1 shows a two group t-test for each of the 40 factor variables between clusters C1 and C2. Of the 40 factors, 13 achieved highly significant cluster differences of *p* < 0.0001, and 11 achieved significant differences with *p* values ranging from *p* ≤ 0.0262 to ≤0.0002. Sixteen tests showed insignificant *p* values. Thus, 60% of the factors manifested significant between-clusters differences, with 32.5% being highly significant. As shown in Table 2, there were no statistically significant differences between the two clusters on the basis of gender, as tested by Chi-square, handedness, also tested by Chi-square, or age in years at time of study, tested by t-test.

**Table 1:** T-test Between Clusters1 (C1) and 2 (C)

FACTOR	C1 Value	C2 Value	T Value	P
1	− 0.1105	0.0346	1.96	ns
2	0.1322	0.2209	−2.65	0.0084
3	−0.2999	0.0101	−3.83	0.0002
4	0.3345	−0.3523	8.19	0.0001
5	0.0442	−0.0152	1.16	ns
6	0.0269	0.3012	−3.86	0.0001
7	0.1229	−0.0814	− 0.37	ns
8	0.1687	0.1176	−2.44	0.0154
9	−0.0398	−0.0032	− 0.52	ns
10	0.1512	−0.1720	3.32	0.0010
11	−0.1320	0.0109	−1.67	ns
12	0.1168	−0.1138	3.16	0.0017
13	0.1116	−0.2466	7.09	0.0001
14	−0.0822	−0.0759	− 0.11	ns
15	−0.1917	−0.6485	5.98	0.0001
16	0.2733	0.1007	1.62	ns
17	−0.1162	− 0.2969	1.93	ns
18	−0.2669	0.0778	−3.55	0.0004
19	−0.1386	0.2480	−3.38	0.0003
20	−0.0413	0.0654	2.23	0.0262
21	0.0318	0.0997	−0.63	ns
22	0.2652	−0.0057	4.95	0.0001
23	−0.0283	0.0393	−0.70	ns
24	0.3523	0.-721	3.48	0.0006
25	−0.2630	0.0748	−4.44	0.0001
26	−0.1591	0.1574	−4.22	0.0001
27	−0.1742	0.3051	−5.59	0.0001
28	−0.0803	0.1718	−2.61	0.0095
29	−0.1108	−0.0029	−1.45	ns
30	−0.0764	0.2491	−3.95	0.0001
31	−0.0431	0.2026	−2.37	0.0185
32	−0.1068	0.0605	−1.86	ns
33	−0.4215	0.2647	−8.36	0.0001
34	0.1884	0.0246	1.91	ns
35	−0.3479	−0.0168	−4.03	0.0001
36	0.2216	0.0621	1.87	ns
37	0.1253	−0.0280	1.79	ns
38	0.1987	−0.1376	5.67	0.0001
39	0.3162	−0.0619	8.50	0.0001
40	−0.2028	−0.1567	0.58	ns

*ns* not significant

**Table 2:** Demographic Differences Between Clusters 1 and 2

A. Gender Difference			
Group	Male	Female	Total
C1	146	23	169
C2	215	46	261
Total	361	69	430
Pearson ChiSquare = 1.228; df = 1; p = ns			
B. Handedness			
Group	Right	Left	Total
C1	159	10	169
C2	254	7	261
Total	423	17	430
Pearson ChiSquare = 2.827; df = 1; p = ns			
C. Age at EEG study			
Group	Mean Age Yrs		Std Dev
C1	4.3769	+/−	2.9645
C2	4.8548	+/−	2.8444
Student’s T = 1.67 p = ns			

*ns* not significant

### Separation of two ASD clusters by two-group DFA

**Fig. 4:**
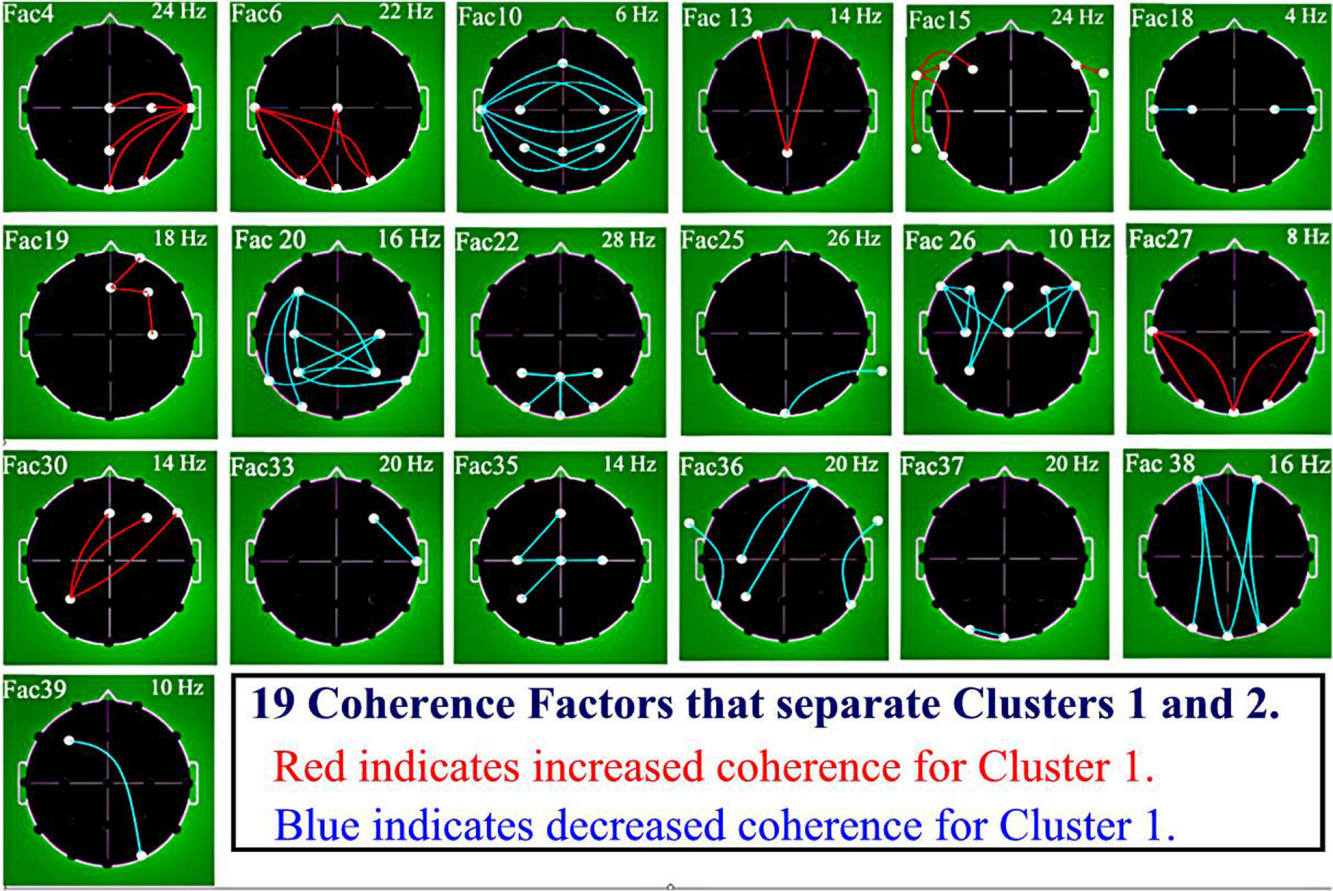
Graphic Representation of 19 Coherence Factor Loadings Used in Separating Clusters 1 and 2. Legend: EEG coherence factor loadings. Heads in top view, scalp left to image left, nose above; Factor number is above heads to left and peak frequency for factor in Hz is above to right. Lines indicate top approximate 15% coherence loadings per factor: Red Lines = increased coherence in Cluster 1; Blue Lines = decreased coherence in Cluster 1. Involved electrodes are shown as white circles. Uninvolved electrodes are not shown; they are blackened-out within the superior scalp area and greened-out for scalp electrodes. Factors are shown in numerical order. See text for factor selection order in discriminant analysis

**Fig. 5:**
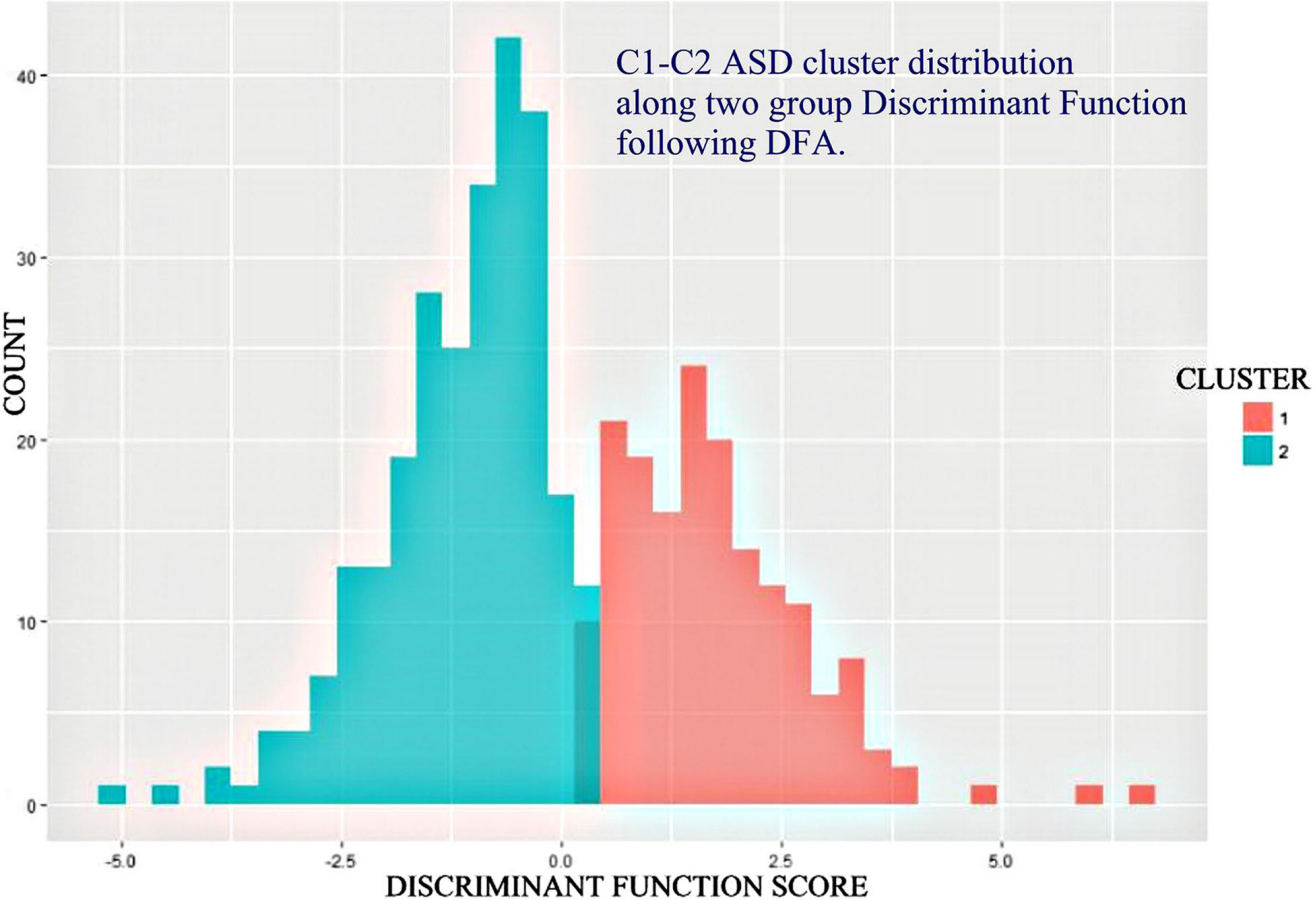
C1 and C2 Cluster Groups Along 2-Group DFA by Discriminant ScoreLegend: C1 and C2 histograms (red = C1, blue/green = C2) with X-axis the 2 group discriminant score. Note minimal overlap. Separation by Wilk’s Lambda is significant (*p* < 0.00001) and overall individual subject classification is approximately 95% correct by jackknifing (see text)

Stepwise DFA (P7M) was performed between ASD clusters C1 and C2 on the initial basis of all 40 Factors as potential discriminating variables. Nineteen factors were selected (Table 3). Coherence loadings on the 19 DFA selected factors are illustrated in Fig. 4. In order to establish the sign, plus for positive, minus for negative, of the differential coherence loading for coherences associated within a given factor (red = positive or blue = negative) three analytic steps were considered for each factor: (1) The sign of loading of coherence upon a given factor at time of factor creation; (2) The sign of the factor loading upon the generated discriminant function variable; and (3) The sign of the C1 and C2 group outcome positions along the discriminant function axis. Note that the C1 - C2 difference involved factors showing both increased (12 red) and decreased (7 blue) coherences. No factor showed a combination of both increased and decreased coherence. Two group classification by Wilk’s lambda was highly successful (0.342; F = 41.4; DF 19,410; *p* < 0.00001). Overall subject classification success was 95.8% directly and 94.7% by jackknifing. Graphic separation between the two cluster groups by the resulting discriminant function is shown in Fig. 5.

**Table 3:** Separation of ASD Clusters 1 and 2 by Discriminant Function Analysis (DFA)

Group	Percent correct	No. of Cases Classified into Clusters	
		C1	C2
Initial Classification Matrix			
C1	94.7	160	9
C2	96.6	9	252
Total	95.8		
Jackknifed Classification Matrix			
C1	92.9	157	12
C2	95.8	11	250
Total	94.7		

Nineteen (19) factor variables were used, presented here in the order of selection: Factors 33, 39, 38, 30, 35, 27, 6, 19, 15, 25, 20, 18, 22, 4, 13, 26, 37, 10, and 36

Five split half replications were performed by DFA between clusters C1 and C2. The population split into test set and training set was performed by means of a random number generator. Results are shown in Table 4. Note that average correct classification of the left out ‘Test Set’ C1 group was 86.36%, and the left out ‘Test Set’ C2 group was 91.79%. Thus, by both jackknifing and by five split half replications there was strong evidence for successful prospective C1/C2 group classification.

**Table 4:** C1 vs. C2, Five Split-Half Replications

Replication 1	
Training Set (*n* = 217)	Test Set (*n* = 213)
C1: 84/89 correct, 94.38%	C1: 72/80 correct, 90.00%
C2: 121/128 correct, 94.53%	C2: 124/133 correct, 93.23%
Replication 2	
Training Set (*n* = 203)	Test Set (*n* = 227)
C1: 80/84 correct, 95.24%	C1: 75/85 correct, 88.24%
C2: 110/119 correct, 92.44%	C2: 130/142 correct, 91.55%
Replication 3	
Training Set (*n* = 228)	Test Set (*n* = 202)
C1: 78/85 correct, 91.76%	C1: 68/84 correct, 80.95%
C2: 133/143 correct, 93.01%	C2: 111/118 correct, 93.07%
Replication 4	
Training Set (*n* = 212)	Test Set (*n* = 218)
C1: 83/86 correct, 96.51%	C1: 71/83 correct, 85.54%
C2: 120/126 correct, 95.24%	C2: 117/135 correct, 86.67%
Replication 5	
Training Set (n = 218)	Test Set (n = 212)
C1: 77/84 correct, 91.67%	C1: 74/85 correct, 87.06%
C2: 129/134 correct, 96.27%	C2: 120/127 correct, 94.49%
	Average Correct Test Set
	C1 = 86.36%
	C2 = 91.80%

### Separation among the two ASD clusters and the CON Group by three-group DFA

**Fig. 6:**
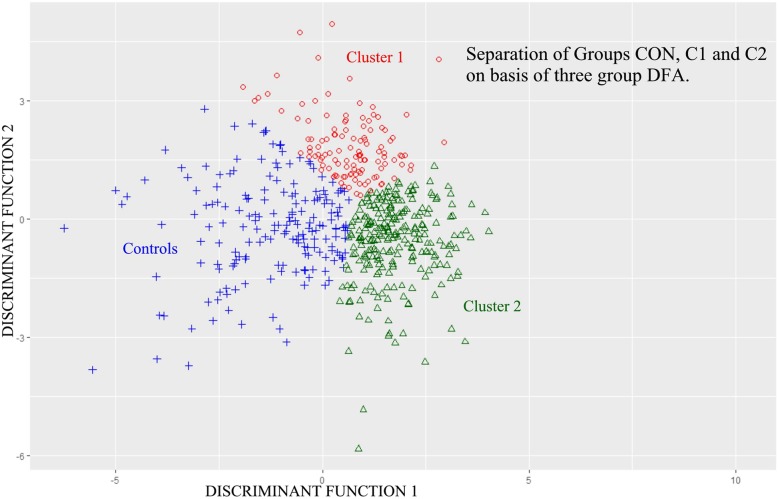
C1, C2 and CON Groups Along 3-Group DFA by Discriminant Score. Legend: C1, C2, and CON group population distributions (red circle = C1, green triangle = C2, blue + = CON) with X and Y axes the two 3-group discriminant function scores. Note minimal populations overlap. Overall and among group separations are significant. There is very significant three group subject classification (see text). Note, hierarchical clustering results, upon which this figure is based, tend to illustrate linear group boundaries (whereas K-means clustering tend to produce more circular or ovoid boundaries [32])

Stepwise DFA (P7M) was performed among ASD clusters C1, C2 and control group CON on the initial basis of all 40 Factors as potential discriminating variables (Table 5). Thirty-one factors were selected for use by DFA. Overall subject classification success among the *three* groups was 87.4% directly and 85.3% by jackknifing. Overall classification and the three separate pair comparisons were all statistically highly significant at *p* < 0.00001 for each of the four analyses. Graphic separation among the three groups (CON, C1, C2) that resulted from this three group DFA is illustrated in Fig. 6. The two discriminant functions served as X and Y axes.

**Table 5:** Separation of ASD Clusters 1 and 2 and the Control Group (CON) by 3 Group DFA

Group	Percent correct	No. of Cases Classified into Group		
		C1	C2	CON
Initial Classification Matrix				
C1	85.8	145	14	10
C2	88.1	11	230	20
CON	87.6	41	28	487
Total	87.4			
Jackknifed Classification Matrix				
C1	81.7	138	16	15
C2	86.6	15	226	20
CON	85.3	49	33	474
Total	85.3			
Thirty-one (31) factor variables were used in the following order: 15, 17, 4, 33, 2, 6, 27, 24, 16, 30,35, 1, 19, 40, 31, 22, 36, 13, 7, 39, 3, 25, 9, 38, 26, 8, 28, 10, 18, 12, 21.				
Significance of Classification Probability:				
Overall	F = 25.61	DF = 62, 1906	*p* < 0.00001	
C1 x C2	F = 17.88	DF = 31, 953	*p* < 0.00001	
C1 x CON	F = 20.77	DF = 31, 953	*p* < 0.00001	
C2 x CON	F = 36.52	DF = 31, 953	*p* < 0.00001

### Passive classification of subjects with Asperger’s syndrome (ASP)

**Fig. 7:**
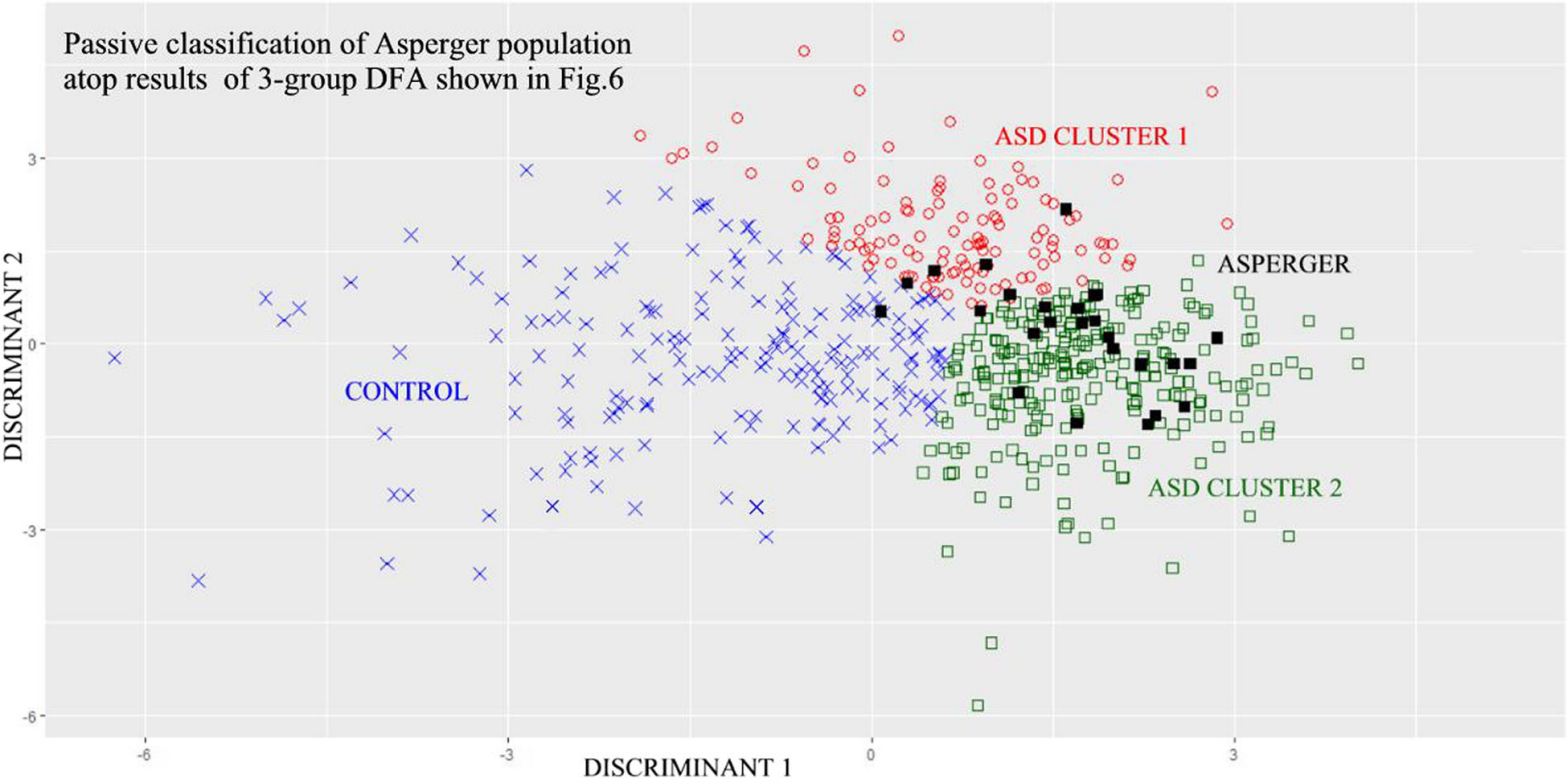
C1, C2, CON, and ASP Groups Along 3-Group DFA by Discriminant Score. Legend: C1, C2, and CON group population distributions as for Fig. 6. Now with *passive* classification of 26 Asperger (ASP) subjects. Note ASP population mostly overlaps with C2 ASD group and nearby regions of C1 group. (red circle = C1, green square = C2, blue x = CON, black square = ASP)

Stepwise DFA was repeated among the three groups C1, C2, and CON on the basis of all 40 Factors. To this three-group population a fourth group of 26 previously studied subjects with Asperger’s syndrome (ASP) [5] was added as input data and set to be *passively classified* by the resulting C1-C2-CON based discriminant functions. The ASP subjects did not participate in the creation of the two discriminant variables. Results demonstrated that 19 of the 26 ASP subjects were passively classified as belonging within the C2 cluster and six within the C1 cluster; of these six subjects two fell into the C1-C2 cluster border zone. One fell within the CON group. These results are illustrated in Fig. 7.

## Discussion

Results show that 430 subjects diagnosed as being on the autism “spectrum” and represented by 40 EEG coherence factors [27], fell into two distinct clusters. These two ‘autism clusters’ statistically differed from one another and, in turn, statistically differed from 554 subject neuro-typical control group subjects, not involved in the clustering process. Notably the 40 utilized EEG coherence factor variables had been objectively derived [27] and a completely objective data-driven variable selection was applied. Furthermore, choice of the optimal cluster number was also objectively determined by use of a relatively recent software package, NbClust [25]. This program was instructed to form up to 15 clusters and to establish the optimal cluster configuration on the basis of the 30 methods [25] included in the program.

Finally, NbClust was run twice, first utilizing the hierarchical clustering technique and second utilizing the other commonly used K-means technique. Both techniques ‘voted’ the two cluster configuration as optimal; the choice was more definitive when hierarchical clustering was used. Thus, the optimal two cluster solution was selected on the basis of objectively derived EEG measures of brain connectivity.

In order to explore the potential clinical significance of the two autism clusters, advantage was taken of a prior study [5] that contrasted the control, ASD, and ASP populations and that had shown that ASP subjects were closer to the ASD population than the neurotypical control population, and also that ASP subjects were statistically separable and distinctly different from the ASD population. For the current study, these previously studied 26 ASP subjects were represented by the 40 EEG coherence factor variables and were utilized to determine whether these ASP subjects would passively fall within one or the other of the newly formed clusters. Notably these ASP subjects had *not* been utilized in the original cluster formation. As Fig. 7 shows, the majority of ASP subjects were within or close to the Cluster 2 domain, which suggests that C2 may be primarily associated with those subjects manifesting Asperger-like behavioral characteristics. It is of note that despite the multiple variable types and differing methods for clustering in the literature, a prominent two cluster distribution of autistic characteristics has been observed repeatedly by others [6, 8, 11, 14, 15].

A significant limitation of the study is the lack of external validation by similarly extensive subject data from other relevant domains, such as neuro-psychological evaluation as well as autistic specific evaluations such as the ADOS [32] or ADI-R [45], MRI [9, 46–49], genetic/epigenetic testing [50–57], and prolonged sleep EEG recordings for detection of epileptiform activity [29, 58–61].

The future establishment of correlations among such additional brain-based data with the EEG coherence-based findings described in this manuscript should be very helpful in further validating the EEG-based subgroups, and should facilitate interpretation of the coherence data by clinicians and scientists alike. The absence of correlative data in the current study does not invalidate the future use of the current data and findings for such correlative studies. For example it was possible in the current study to insert 26 Asperger’s patient data within the three group discriminant analysis described here. As also previously reported, autistic patients could be additionally classified as also having attention issues using a discriminant, developed on a different population with attention disorders. Thus it was possible to explore attention disability within autism [40] by means of EEG data. Future studies utilizing the current study’s results would only require that subjects have waking EEG data. Such studies are anticipated.

It is also important to clarify that the current demonstration of two neurophysiological clusters within the autism spectrum does not preclude the possibility of further, relevant autism subdivisions. For example in the future, as the neurodevelopmental characteristics of Cluster 1 and 2 are explored, there may prove to be additional clinically relevant sub-populations within or even across these two clusters. .

## Conclusion

Objective brain derived EEG coherence factor data strongly support the proposition that the autism disorder should not be seen as a continuous spectrum [1] but likely is formed from *at least* two distinct subpopulations. This is important since the ‘spectrum versus clusters’ issue goes beyond academic taxonomy and has a number of real world consequences: (1) For example, moving Asperger’s syndrome subjects into the autism spectrum disorder allows some US schools to develop and offer a *single* autism educational program that is oriented towards management and teaching of the ‘typical’ autistic child of limited verbal ability, who may present with behavioral issues. This may leave out or otherwise disadvantage children with Asperger’s Syndrome, who present with specific and often different behavioral and educational issues altogether and typically profit from more individually-tailored education. (2) On the other hand, in clinical autism research (e.g., neuro-behavioral evaluations, MRI based studies) it is often much easier to recruit and successfully study subjects with Asperger’s Syndrome or others who are high-functioning. However, results of such studies may be inappropriately generalized as findings characteristic of the entire autism spectrum. (3) The multiple different findings resultant from genetic and epigenetic studies of autism [54, 62–65] also contradict a unitary perspective on autism. Important correlational findings may be lost when all autism is treated as a single entity. It prevents identification of distinct subgroups based upon clinical insights, and/or neurobehavioral parameters, and/or direct brain parameters (as from MRI and EEG).

In addition EEG, a classic and relatively inexpensive non-invasive test, is found to be reasonably well tolerated by children with various forms of ASD and should be considered for inclusion in future studies of autism and of other neurobehavioral disorders [5, 27, 40, 66, 67].

As previously discussed [27] the authors believe that in clinical practice diagnosis of ASD should follow the DSM-5 criteria, and should be made by clinicians with special training in this area (e.g., neurologists, psychiatrists, psychologists) by use of readily available assessments such as the ADI-R [45]. EEG coherence study data may best serve as adjunctive, confirmatory, and/or exploratory information. At this point they are especially useful regarding the discovery of clinically relevant autism sub-populations.

## Abbreviations

ADD: Attention deficit disorder
ADHD: Attention deficit hyperactivity disorder
ADI-R: Autism diagnostic interview - revised
ANOVA: Analysis of variance
ASBDC: Autism spectrum disorder checklist
ASD: Autism Spectrum Disorder
ASP: Asperger’s syndrome
BCH: Boston Children’s Hospital, an HMS affiliated teaching hospital
C1: Cluster one
C2: Cluster two
CON: Neurotypical control group
DFA: Discriminant function analysis
DNL: Developmental neurophysiology laboratory at BCH
DSM: Diagnostic and statistical manual (of Mental Disorders)
EEG: Electroencephalogram, electroencephalography
FFT: Fast fourier transform, used for spectral analysis
HMS: Harvard Medical School (Boston, Massachusetts, USA)
IRB: Institutional review board of BCH
PCA: Principal Components Analysis
PDD-nos: Pervasive developmental disorders – not otherwise specified
